# Supplementary material for: Patterns of Intron Gain and Loss in Fungi
Source: PLoS Biol. 2004 Nov 30;2(12):e422. doi: 10.1371/journal.pbio.0020422 (PMC532390; doi:10.1371/journal.pbio.0020422)
Supplement: Table S1 — Also available at http://genes.mit.edu/NielsenEtAl/. (4.3 MB ZIP). [file pbio.0020422.st001.zip › NielsenEtAl/html/41.html]

AN4967.1.NCU04743.1.MG02486.1.FG09516.1


```
 CLUSTAL W (1.82) Multiple Sequence Alignments - Introns Inserted


Sequence 1: NCU04743.1	854 aa
Sequence 2: FG09516.1	771 aa
Sequence 3: MG02486.1	856 aa
Sequence 4: AN4967.1	757 aa
Alignment Length: 939 aa
Number Identitical Residues: 300 aa
Alignment Score (without introns) 15645


MG02486.1 	MHIL~VVNDDGPPSSQSSPYVHTLVRALQGAGHTVS~VCLPHTQRSWIGKAHLIGQTVRP
NCU04743.1	MHIL~IVNDDGPPSAHSSPYVHSLVRDLQAAGHTVS~VCLPHTQRSWIGKAHMIGQTVKP
FG09516.1 	MHIL~VTNDDGPPSPHSSPYVHCLIQQLQQAGHTVS0----------------------P
AN4967.1  	MHIL0VVNDDGPPSNESSPYVHSFVHTLQSAGHTVS~VVLPHQQRSWIGKAHLIGAAVKP
          	**** :.******* .****** ::: ** ******    .  . :  ..:   . :  *

MG02486.1 	TYYRPPPLPAN----PAGLIFDKDVPE------SEGSTHKRPTTTAD-EEWVLVDGTPAS
NCU04743.1	LYYRPPPASSPAAGLTTALVPSSEKPEQTVNVTDHGSVHLRPSTVPGTEEWILVDGTPAS
FG09516.1 	LYYRP-----------SSVVHGDESP---------GTTHHRPSPSGDVEEWVLVDGTPAS
AN4967.1  	TYFRP------------GTLHKDDGTT---------HEYPRGDNDPDGDEWILINSTPAS
          	 *:**            . :  .: .            : *     . :**:*::.****

MG02486.1 	CVQIGLHHMFTDRGKVDLVLSGPNYGRNTTALFALSSGTLGGALEGAACKVPSIALSYAF
NCU04743.1	CVQIGLYHFFQDRGPVDLVVSGPNYGRNTTAVFALSSGTLGGALEAAVCKRRAIALSYAF
FG09516.1 	CVQIGLHHFFQDKGPIDLVVSGPNYGRNTTAVFALSSGTLGAALEAAVCQKKSIALSFAF
AN4967.1  	CVQIGLYHYFQDRGPVDLVVSGPNYGRNTTALFAMSSGTIGAAMEGAACGKRSIALSYAF
          	******:* * *:* :***:***********:**:****:*.*:*.*.*   :****:**

MG02486.1 	FTRNHDPEIVAGASRHAVRVVEALVKQWPQDGSVDLYSVNIPLVEGVEKARTLWTPMLQN
NCU04743.1	FNRNHDPAIITKASRQSVRVIEALWKQWPTDGSVDLYSVNVPLLEGLEEGKVLYTPMLQN
FG09516.1 	FTRNHDPVIIEAACRRSVKVIENLYKQWPTDGSADLYSVNVPLIEGLENNKAIWTNVLQN
AN4967.1  	SSRNHDPVIIAEASRHSVRVIEYLAKNWDEG--VDLYSVNVPLEPGVSESKVMYTEMLDN
          	 .***** *:  *.*::*:*:* * *:*  .  .******:**  *:.: :.::* :*:*

MG02486.1 	YWADGGCFTAVEGAGDEDEDETEERIRQGAGGEAALSSGAQNGKAAADGGEGTAHTRTHK
NCU04743.1	YWGAGSCFEEVEGSVD-GEEVDEERIREGGGADAETGDGGGGLEVGDGKRDGREGLHTHK
FG09516.1 	YWREGGCFQEIEGEAG-DENEEEERIREGVGG-----------EVDDAARPSSRKGHTHK
AN4967.1  	RWSSGSCFDAVDAEVPVENPEQREQTLRDQEEKLE-----EDPTANPNAKSGRKSRIAHK
          	 *  *.**  ::.     :   .*:  ..   .        .  .      .     :**

MG02486.1 	HFKWQPRFTDVYKSVEDAPPGNDGWAVKEGYTS2VTPLKANFWQAATHLHG-QDLKL2PV
NCU04743.1	HFKWSPRFTDVYKSVEEAPPGNDGWAVKEGHTS2VTPLKANFWNTAENLHG-KELQL2PP
FG09516.1 	HFKWAPKFTDVYKSVEESEPGNDGWAVKEGLTS2ITPLKANFMLGAGELFNQKEFEL2D-
AN4967.1  	HFIWAPKFTDVYRSVEESAPGNDGWTVKEGMTS2VTPLKANFMHTPG-IKG--EIKL2S-
          	** * *:*****:***:: ******:**** ** :*******   .  : .  :::*   

MG02486.1 	LESHEGTDTLPIRSAAGIGAAATEQSINKTSPAGDKGAYDSQDTGKLHALIAYDDPYVQP
NCU04743.1	LE----------TPSAIKTESTTTLPIRNSTTTANSKDH-------LYALIDYQDAYVQP
FG09516.1 	-------------SGSVANQSTQEMALR------PKGPS-------IQAVISYEDAYVQP
AN4967.1  	----------------------------------DNEEP------AFYSIVDCDDPYVQE
          	                                   .          : :::  :*.*** 

MG02486.1 	LIRDALGSLLPSDKYCLVDAPEQQPEDKDNPVSLASLIPGDGTKALQIASYEAIDFDYAA
NCU04743.1	LILSAIEKLLPSSSYTLLPSPF-TSENKEPEIHLSTLLPSEDAKVLQITPYETIDFDHAM
FG09516.1 	LILSALNSIFPEGVFNVITEVP-ESD----EPALAKIVPSE-ENILQITAYESIDFEYAG
AN4967.1  	LVEQALRFSMGSRCR---------------SVSSISELPSRSTPVFQYREYERLDFEHAM
          	*: .*:   : .                       . :*. .   :*   ** :**::* 

MG02486.1 	EHQNSIVINSYKFRKALIRKHFLATTVENWVIKHPNSILARHVKPSTSFEVDYAEFLDDA
NCU04743.1	SHPATTLINSYIIRKALIRKHFLSSTVENWVAKHPTSALKTHVKRAEAFEVDYAEFLDDS
FG09516.1 	SHERTTLINSYMIRKALIRKHFLSTTVDHWVAKHPESVLKTHIKRSEAFEVDFAEFLDDA
AN4967.1  	TNPTTSLINAYIIRKALIRKHYLANTVSNWVTKHPESVLAKHVKVSVDFELDYAEFLDDA
          	 :  : :**:* :********:*:.**.:** *** * *  *:* :  **:*:******:

MG02486.1 	LVEAWDLRASLENDE---------KREWWILKPSMSDRGQGIRLFSTMEELQGIFDEWDV
NCU04743.1	LVEAFDLRASMEKNDQLIAEGKEREVEWWILKPSMSDRGQGIRLFSTMEELQSIFDSWEV
FG09516.1 	LVEAFDLRESMDRNE---EQSDPSSKEWWILKPGMSDRGQGIKLFSSMDELQNIFDIWEE
AN4967.1  	LLEAYELRECFEENE----SRPDSEKVWWILKPGMSDRGQGIRLFNSEDQLREIFEEWEP
          	*:**::** .::.::    .    .  ******.********:**.: ::*: **: *: 

MG02486.1 	-----DTDDEDE-------------------------EADGIMAAHLRHFVAQPYIDPPL
NCU04743.1	ESDSEDEDDDDASSSNADNTSSNADDTSDAGSDHGDGNGNGINTSHLRHFIAQPYIHPPL
FG09516.1 	--DQPDTDDEDEVADNDN-------------------DGDGITTSHLRHFVAQPYIHPPL
AN4967.1  	DSEDEEEDECGEDDGNDE---------------TDKAATSGVVTSQLRHFIAQPYIDPPL
          	.:.. : *: .   .. .                ...  .*: :::****:*****.***

MG02486.1 	LLPGD-RRKFHIRTYVLCVGAMKVYVYREMLALFAGREYRAPSSDE--------------
NCU04743.1	LLPELSNRKFHIRVYVLAIGALKVYVYKDMLALFAGVPYTSPTTSSSSDPDSNPDSEPAG
FG09516.1 	LVDGE-KRKFHIRTYVMCSGSLDVWVYKHMLALFAGKPYTAPADAP--------------
AN4967.1  	LLPSLNNRKFHIRTYVLATGSLKVYVFKEMLALFASKPYVSPTSQNNETEDS--------
          	*:   ..******.**:. *::.*:*::.******.  * :*:   ..  .:        

MG02486.1 	-GDLDAHLTNTCLQNETTAASATATAADIATSDAKDGEAPPPPLVRKFWDLPEVLV--EG
NCU04743.1	ELDLSAHLTNTCLQTYLSPNAA-------------------ENSVHRFWDLSSLSP--TH
FG09516.1 	-EDIESFLTNTCLQD--SPN---------------------ENTVRRFWDLP--------
AN4967.1  	IADLTRHLTNTCFQDKSLPE---------------------SETVRRFWCLPSIPPPNTH
          	  *:  .*****:*    .                         *::** *..   ..  

MG02486.1 	RSDVSKGSIFTQICEATGAVFEAAARGMPMHFSPLANAFEVFGVDFLVDAQG-----VAW
NCU04743.1	FPQSKAENIWDQICEVTGDLFEAAARGMMIHFQPMEQAFEVYGLDFLVDADDESGRNTAW
FG09516.1 	LSNDMRDDIFRQICDVTGEIFEAAAKAMPIHFQTMPNAFEVYGLDFMVDAQG-----TAW
AN4967.1  	LTATWKEDIYEQICAVTGELFTAAARGMMIHFQTMPNAFEVFGVDFLVDDTG-----NVW
          	 .     .*: *** .** :* ***:.* :**..: :****:*:**:**  .      .*

MG02486.1 	LLEVNSFPDFKQTGDELRDDVVAELWRQVLRLAVVEGGPLRLGGGKGDRDDEGHGDATGA
NCU04743.1	LLEVNAFPDFKQTG-ELKG-VVGGFWEGVVREAVGG----FVGVQ----KGDSDEKMRLV
FG09516.1 	LLEVNAFPDFKQTGGDLKE-IVSGFWKGVMRHGVAP----FFGIESKIRDQEGAEDMVPV
AN4967.1  	LLEVNAFPDFGQTGEELRDVVVGGLFKGVIGVAVKG----FFGEE----GKTEENGMRLV
          	*****:**** *** :*:  :*. ::. *:  .*       .*                .

MG02486.1 	TT~KTLS---------AAAAAGDG~KMVLVKDVDLGKEGLLGFLG
NCU04743.1	R-~---------------------~------DVDLGRRW------
FG09516.1 	RK0SIKGNQSPPYHNLAATREFKV1ALKKDNNSQLGLRSNYTATM
AN4967.1  	A-~---------------------~------ELDLGRKN------
          	                               : :** .
```
